# The effects of *pprI* gene of *Deinococcus radiodurans* R1 on acute radiation injury of mice exposed to ^60^Co γ-ray radiation

**DOI:** 10.18632/oncotarget.13893

**Published:** 2016-12-10

**Authors:** Ting-ting Chen, Wei Hua, Xi-zhi Zhang, Bu-hai Wang, Zhan-shan Yang

**Affiliations:** ^1^ Department of Oncology, The People’s Hospital of Subei, Yangzhou, China; ^2^ School of Radiation Medicine and Protection, Medical College of Soochow University, Suzhou, China

**Keywords:** bacteria, gene therapy, radiation protection, pprI gene, in vivo gene electroporation, Pathology Section

## Abstract

The role of the *pprI* gene from *Deinococcus radiodurans* R1 in therapy of acute radiation injury of a mammalian host was investigated. We injected a plasmid containing the *pprI* gene into the muscle of mice exposed to total 6Gy of ^60^Co γ-ray radiation. After injection, we used in vivo gene electroporation technology to transfer the *pprI* gene into the cell. We found the PprI protein was expressed significantly at 1 d after irradiation, but there was no expression of *pprI* gene 7 d post-irradiation. The expression of *pprI* gene evidently decreased the death rate of mice exposed to lethal dose radiation, significantly relieved effects on blood cells in the acute stage, shortened the persistence time of the decrease of lymphocytes, and decreased the apoptotic rates of spleen cells, thymocytes and bone marrow cells. The expression of Rad51 protein in the lungs, livers, and kidneys was significantly higher in the mice treated with the *pprI* plasmid after irradiation. However, there were no obvious differences for Rad52 protein expression. We conclude that the prokaryotic *pprI* gene of *D. radiodurans* R1 first was expressed in mammalian cells. The expressed prokaryotic PprI protein has distinct effects of the prevention and treatment on acute radiation injury of mammal. The effects of radio-resistance may relate to expression of Rad51 protein which is homologous with RecA from *D. radiodurans*.

## INTRODUCTION

*Deinococcus radiodurans* R1 is one of the most radiation resistant organisms on the earth [1]. It can tolerate many abiotic stresses including ionizing radiation, UV radiation, desiccation, mitomycin C, and hydrogen peroxide [2, 3]. Many mechanisms have been proposed to explain this extreme resistance to radiation [3-10], such as efﬁcient DNA repair processes, efﬁcient protection of proteins against oxidation, and a more compact structure of the nucleoid. Many previously unknown functional protein [11], have been found to play important roles in *D. radiodurans* such as PprA [12], RecA [13], PolA [14], RecN [15]. The protein PprI (also called IrrE) is one of the repair proteins in *D. radiodurans*. It is encoded by gene DR_0167, and plays an important role in stress resistance. A strain deleted for this gene is sensitive to ionizing radiation, UV radiation, desiccation, and mitomycin C [16]. The *pprI* gene can promote *recA*, *pprA* genes in cells to participate the DNA damage recovery gene expression, and the PprI protein can incentive *recA* and *pprA* to increase genetic transcription after radiation, accordingly, it accelerates DNA damage repair which caused by ionizing radiation [17].

Interestingly, introduction of PprI has been shown to enhance the radioresistance and salt-resistance of *E.coli* and increase the salt resistance of Brassica napus [18]. At least in *E.coli*, PprI functioned as a global regulator initiating a complex transcriptional cascade [19]. *D. radiodurans* is a Gram-positive bacterium belonging to the phylum Deinococcus-Thermus; phylogenetically very distant from mammals. *D. radiodurans* and mammal differ in genetic constitution, protein expression, and codon preference. The mammalian cells do not contain the *pprI* gene or homologous analogues. We asked if transfer of the *D. radiodurans pprI* gene to mammals would express and confer anti-radiation protection. We constructed an eukaryotic plasmid, pCMV-HA-*pprI* (patent number: 200910003512.2, 2009-07-29, China) and transferred the plasmid to the muscle of mice by *in vivo* electroporation to study the preventive and therapeutic effect of *pprI* gene in mammalian cells after γ ray radiation.

## RESULTS

### The expression of PprI protein in the muscle of mice

**Figure 1:**
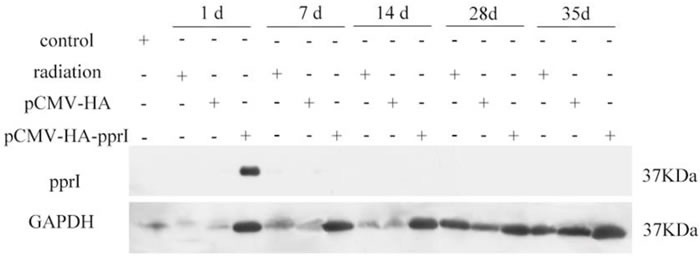
The expression of PprI in the muscle of mice in control group, radiation group, pCMV-HA transfection group, or pCMV-HA-*pprI* transfection group at different amounts of time after radiation GAPDH was used as a loading control.

After the transfection of plasmid, we measured protein levels in the muscles by western blot. We assayed 5 mice in each group on the 1st, 7th, 14th, 28th and 35th days after radiation. The protein PprI expressed highly on the 1st day after radiation, demonstrating that the plasmid of pCMV-HA-*pprI* transferred to the mice successfully, but we didn’t detect the protein in mice on the 7th, 14th, 28th and 35th days after radiation (Figure 1). Glyceraldehyde-3-phosphate dehydrogenase (GAPDH) was used as a loading control.

### The mortality of mice

**Figure 2:**
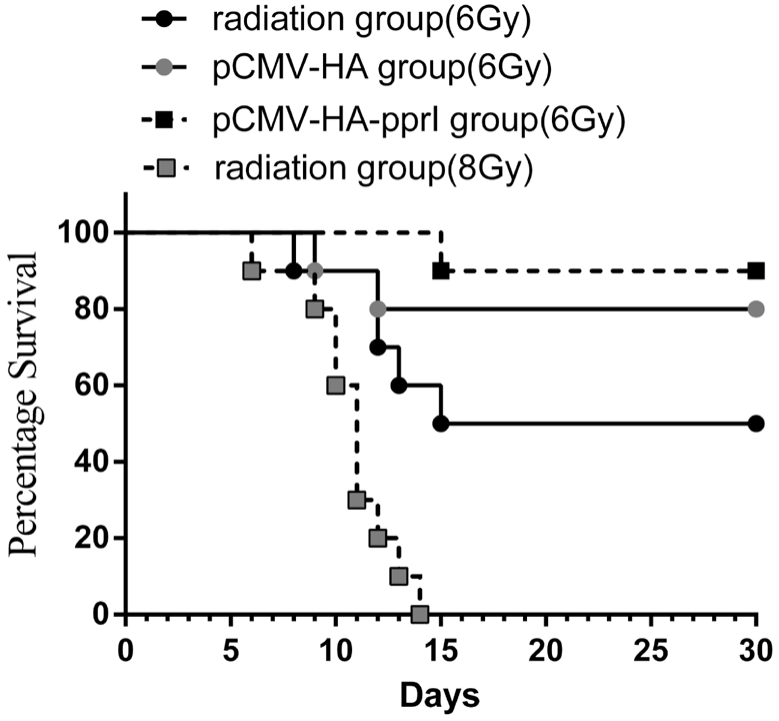
The morality of mice in 6 Gy radiation group (*n* = 10), 6Gy radiation pCMV-HA transfection group (*n* = 10), 6Gy radiation pCMV-HA-*pprI* transfection group (*n* = 10) and 8Gy radiation group (*n* = 10) after radiation Kaplan-Meier survival curves presenting mean survival of each group over time. Curves were analyzed using the log rank test. A significant difference in mortality was observed between the 6 Gy radiation group and 6Gy radiation pCMV-HA-*pprI* transfection group. (*P* < 0.05).

As radiation dose increased, mortality of mice increased. In preliminary experiments, we tested 8Gy radiation but found that this resulted in death of all the mice (Figure 2). For this experiment, absorb dose of 6Gy with ^60^Co γ-ray was used. To assess whether the *pprI* gene can treat radiation injury, the mice of 6Gy radiation group, 6Gy radiation pCMV-HA empty vector transfection group, or the 6Gy radiation pCMV-HA-*pprI* gene transfection group were monitored for 30days. After 6Gy γ-ray radiation, the mortality rate of pCMV-HA-*pprI* transfection group (10%) was lower than radiation group (50%) and the empty vector, pCMV-HA group (20%). (Figure 2).

### The change of the peripheral blood

**Figure 3:**
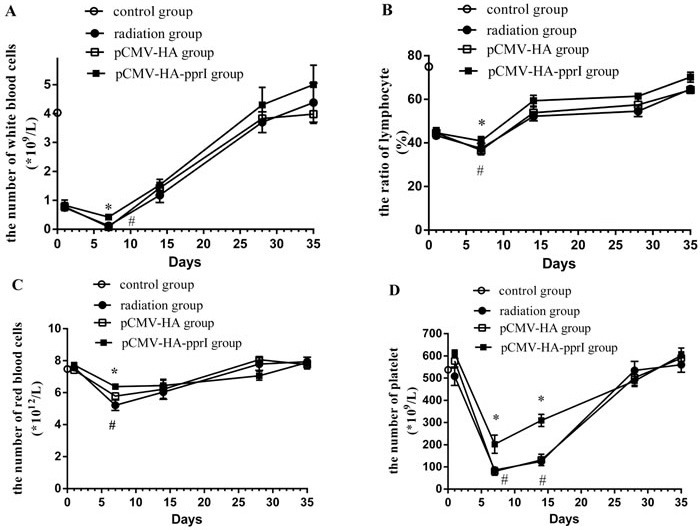
The change of the peripheral blood in the control group, the radiation group, the pCMV-HA transfection group and the pCMV-HA-*pprI* transfection group after radiation Data are means ± S.E.M (standard error of the mean). from 3 replicate experiments, each with 5 mice per group. Error bars indicate S.E.M. Data were analyzed by one-way ANOVA with SNK test. The asterisk (*) indicates a significant relative to the radiation group. The (#) indicates a significant difference relative to the transfer pCMV-HA group. **A.** The change of white blood cells. **B.** The change of the lymphocyte-to-white blood cell ratio. **C.** The change of red blood cells. **D.**) The change of platelet numbers.

We found that after radiation, the white blood cells and the lymphocyte-to-white blood cell ratio of the radiation group, the transfer pCMV-HA group and the transfer pCMV-HA-*pprI* group all obviously decreased on the 1st day after radiation, and the minimum value was on the 7th days after radiation (Figure 3). This indicated that the white blood cells and the lymphocyte-to-white blood cell ratio were sensitive to radiation. Then, the lymphocyte-to-white blood cell ratio began to recover on the 14th days. The white blood cells became normal on the 28th days, but the lymphocyte-to-white blood cell ratio remained low. Compared to the radiation group and the pCMV-HA group, the number of the white blood cells and the lymphocyte-to-white blood cell ratio of the transfer pCMV-HA-*pprI* group was significantly higher, especially on the 7th days (*P* < 0.05). In addition, we found that the red blood cells and the platelet numbers of the three groups were not obviously decreased on the 1st day, but on the 7th days, they were all obviously decreased, and then began to recover from the 14th days. Compared to the radiation group and the pCMV-HA group, the number of the red blood cells of the transfer pCMV-HA-*pprI* group was higher on the 7th days (*P* < 0.05). Additionally, the platelet number of this group was higher on the 7th days and the 14th days (*P* < 0.05). This demonstrated that *pprI* gene electroporation *in vivo* could alleviate the decrease of white blood cells, red blood cells, and platelet, and advance the recovery of the lymphocyte-to-white blood cell ratio after radiation.

### The apoptosis of spleen cells, thymus cells, and bone marrow cells of mice

**Figure 4:**
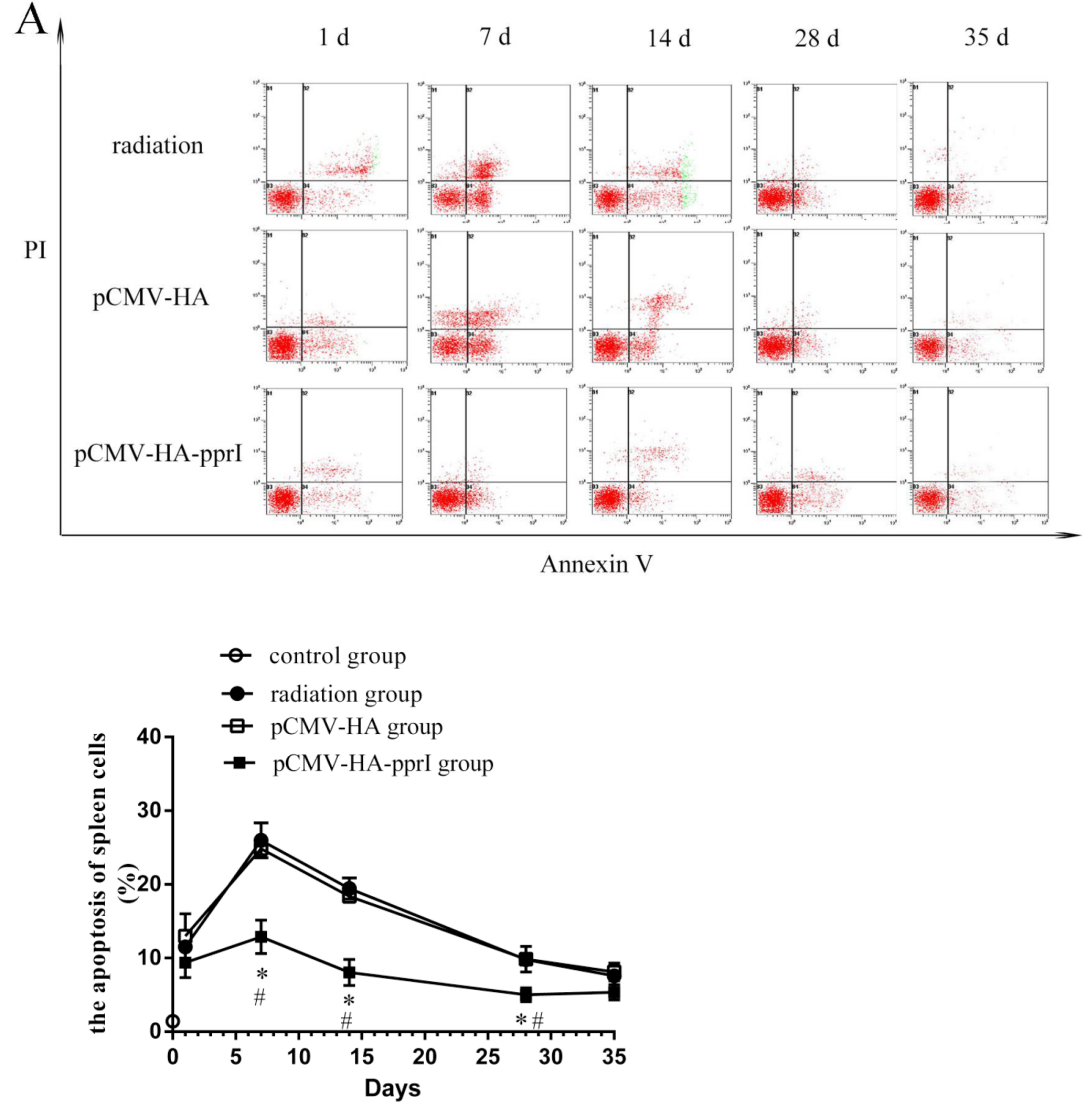
The apoptosis of cells in control group, radiation group, pCMV-HA transfection group and pCMV-HA-*pprI* transfection group after radiation Cell apoptosis of **A.** The spleen cells, **B.** The thymus cells, **C.** The bone marrow cells were measured by annexin V-FITC/PI staining and analyzed with flow cytometry. Horizontal and vertical axes represent labeling with annexin V-FITC and PI, respectively. Data are means ± S.E.M. from 3 replicate experiments, each with 5 mice per group. Error bars indicate S.E.M. Data were analyzed by one-way ANOVA with SNK test. The asterisk (*) indicates a significant relative to the radiation group. The (#) indicates a significant difference relative to the transfer pCMV-HA group.

We found that the apoptosis of spleen cells, thymus cells, and bone marrow cells of the three groups were higher after radiation (Figure 4). Spleen cells, thymus cells, and bone marrow cells were sensitive to radiation. Apoptosis of the three kinds of cells was highest on the 7th days, and then became to recover. Significantly, the apoptosis of spleen, thymus, and bone marrow cells of the transfer pCMV-HA-*pprI* group were lower than the other groups on different days post-radiation (*P* < 0.05), demonstrating a protective effect of the *pprI* gene.

### The expression of Rad51 protein

**Figure 5:**
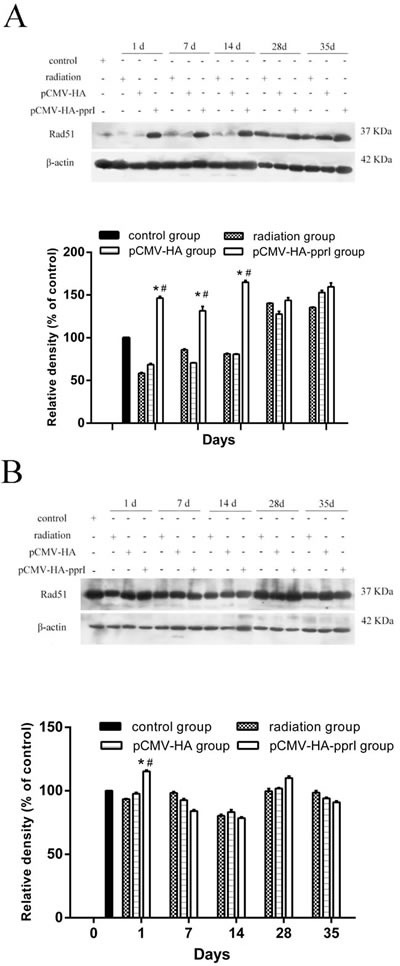
Comparison of the differences in expression of Rad51 in control group, radiation group, pCMV-HA transfection group and pCMV-HA-*pprI* transfection group on different days after radiation The activity of Rad51 and β-actin **A.** In the lungs of mice, **B.** In the livers of mice, **C.** In the kidneys of mice were examined by western blot assay. β-actin was used as an internal control for relative quantification of gene expression. The relative density of the above proteins was normalized to β-actin, which was determined by densitometric analysis. The values represented are means ± S.E.M. from 3 replicate experiments, each with 5 mice per group. Error bars indicate S.E.M. Data were analyzed by one-way ANOVA with SNK test. The asterisk (*) indicates a significant relative to the radiation group. The (#) indicates a significant difference relative to the transfer pCMV-HA group.

We measured levels of Rad51 protein in the lungs, livers, and kidneys, and found higher expression in mice from the pCMV-HA-*pprI* group, especially on the 1st day after radiation (*P* < 0.05) (Figure 5). In the lungs of mice, this effect persisted 7 and 14 days after radiation, with the Rad51 protein levels remaining elevated (*P*< 0.05). After 28 days, this effect was lost, and the Rad51 levels were indistinguishable from those of the other treatment groups. But, in the livers and kidneys of mice, this effect didn’t persist after radiation. The reason was not clear. We supposed that it might have same relationships with radiation pneumonitis, which occurred in the early stage after radiation and produced many cytokines. [20, 21]

### The expression of Rad52 protein

**Figure 6:**
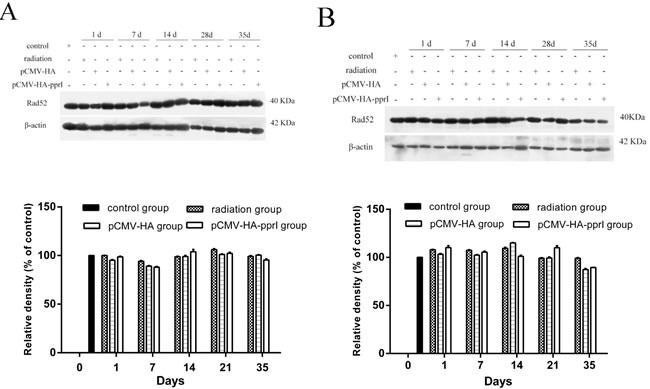
Expression of Rad52 in control group, radiation group, pCMV-HA transfection group and pCMV-HA-*pprI* transfection group on different days after radiation The activity of Rad52 and β-actin **A.** In the lungs of mice, **B.** In the livers of mice, **C.** In the kidneys of mice were examined by western blot assay. β-actin was used as an internal control for relative quantification of gene expression. The relative density of the above proteins was normalized to β-actin, which was determined by densitometric analysis. The values represented are means ± S.E.M. from 3 replicate experiments, each with 5 mice per group. Error bars indicate S.E.M. Data were analyzed by one-way ANOVA with SNK test. But there was not any significances.

Rad52 protein is also important for homologous recombination, and is conserved in eukaryotes. We also measured Rad52 levels in lungs and livers and found no obvious differences between treatments (Figure 6).

## DISCUSSION

The *pprI* gene acts as a switch gene in *D. radiodurans* [22]. When the *D. radiodurans* PprI protein was expressed in *E. coli*, the complemented *E. coli* strain showed an increase of approximately 1.6-fold radio-resistance with a high dose (50 and 100 Gy) of gamma irradiation. Expression of PprI protein in *E. coli* significantly enhanced the scavenging ability of free radicals by inducing the enzymatic activity of KatG and increasing KatE activity 1.5 to 2 times in the exponential growth phase and 2.5 to 3 times in stationary phase [17]. These results indicate that the exogenous protein PprI promotes DNA repair and protection pathways, and it enhances the radioresistance of *E. coli* [17]. PprI functions as a global regulator initiating a complex transcriptional cascade in the heterologous *E. coli* host, up-regulating transcription of at least 210 genes after radiation, including 21 DNA repair and replication-related genes [19]. PprI was found to speciﬁcally bind the promoters of recA and pprA *in vitro* but not bind nonspeciﬁc double-strand DNA, suggesting a direct effect of PprI on increasing transcription of these genes. DNA-binding activity is essential for PprI to program the DNA repair process and cellular survival of *D.radiodurans* in response to radiation damage [23].

*D. radiodurans* and mammals are very different, especially in genetic constitution, protein expression and codon preference. We are unaware of other work testing the application of *pprI* in mammalian systems. In this study, we transferred a eukaryotic plasmid of pCMV-HA-*pprI* to mice by *in vivo* electroporation transfection, an efficient tool for delivery of genes into mammalian cells and gene therapy [24, 25]. This method uses electrical fields to create transient pores in the cell membrane that allow the entry of normally impermeable macromolecules into the cytoplasm [26]. And this method increases the level of transfection ten-fold as compared to the direct administration of a “naked” plasmid into muscle [27-29]. Nowadays, it has been commonly used for introducing genetic material to cells [27], and for delivering drugs across the cell membrane. However, there are also a lot of factors which can influence the transfection efficiency of this method, such as electric pulses [30], reactive oxygen species [31], inhibitors of endocytosis [32], and so forth. In this study, we confirmed successful transfer of the plasmid encoding pCMV-HA-*pprI* and found expression in mice 1 day after radiation, but not subsequently. The reason of later loss of the plasmid might be that the immune response to transgenic products hampered the lasting gene expression [33].

The mortality rate of transfer pCMV-HA-*pprI* group was obviously lower than radiation group, this result suggested that PprI can reduce the mortality of mice irradiated.

Hematopoietic tissues are one of the most sensitive to radiation [34]. Peripheral blood cells are critical for immune response [35]. After acute irradiation with γ-ray, blood cell counts decrease and can induce inflammation and internal hemorrhage or even lead to death [36]. Lymphocytes are the most sensitive blood cell type. These cells mediate the adaptive immune response, and have life spans of weeks to years. Lymphopenia has been used as an indicator of the systemic effects of radiation exposure. Here, we found that the numbers of white blood cells, red blood cells, platelet cells, and the lymphocyte-to-white blood cell ratio all decreased after radiation, indicating that there was serious injury to the hematopoietic system after γ-ray irradiation. The group with the *pprI* gene exhibited less damage, indicating that the gene has some protective effect on the γ-ray radiation damage of mice.

Cell apoptosis induced by radiation is regulated by a complex balance between pro-apoptotic factors caspase-3, c-caspase-3 and Bax, as well as anti-apoptotic factors, and the Bcl-2 family [37, 38]. We measured apoptosis of spleen cells, thymus cells, and bone marrow cells, and found significant increases after γ ray radiation and an altered leukocyte and the lymphocyte-to-white blood cell ratio in peripheral blood. Interestingly, in this result, we found the apoptosis of spleen, thymus, and bone marrow are not in the same extent, the reason may be because that the different tissues contain different kinds of lymphocyte cells, their radiosensitivities are different. However, the apoptosis in tissues from mice that received the pCMV-HA-*pprI* group was obviously lower consistent with a model in which PprI protein confers a protective effect on mice after acute radiation injury. PprI protein could be used as an effective radio-protective agent in the future.

Although we see that *pprI* can reduce the consequences of radiation, its mechanism of action remains poorly understood. Various types of DNA damage are caused by radiation, and one of the most severe are DNA double-strand breaks (DSBs) [39, 40]. After radiation, *D. radiodurans* will produce over thousands of DSBs in each cell, but the bacteria still survive because of its efficient and precise reassembly of hundreds of short DNA fragments by an as yet, unresolved mechanism [7]. In *E.coli* and mammals, there are two ways to repair the DNA double-strand breaks: the major pathway of homologous recombination repair and the lesser used non-homologous recombination repair [41]. In *E. coli*, homologous recombination is mainly performed by the multifunctional RecA protein; in eukaryotes, this process is performed by a variety of proteins, including Rad51, Rad52, Rad55, Rad57, and other proteins. In *D. radiodurans*, RecA protein also plays a central role in DSB repair, essential for homologous recombination repair [42, 43]. The RecA protein of *D. radiodurans* has homology to both the RecA protein of *E. coli* and the Rad51 protein of eukaryotes. The identity of amino sequence of the RecA protein of *D. radiodurans* and *E.coli* is 55%; the HsRad51 and RecA protein of *D. radiodurans*, have 28% identity and 43% similarity, and the homology between hsRad51 and RecA of *E.coli* ~55%. The Rad51 protein plays an important role in the homologous recombination repair, is expressed in the meiotic cells and mitotic cells, is the only protein factors that can catalytic homologous pairing in homologous recombination repair of mitotic cells [44]. Rad52 protein is also important for homologous recombination [45], and is conserved in eukaryotes [46]. In the mitosis, Rad52 protein first recognizes the ends of the DNA molecule in the presence of a DNA double-strand break, and then a complex formed by Rad51, Rad55 and Rad57 molecules binds to the Rad52 molecule, accordingly, they initiate the exchange of homologous chains. This series of proteins through the interaction between molecules to form a complex, completing DNA double-strand break repair together, and this structure can be called “recombinant”. In the absence of Rad52, almost all forms of homologous recombination in humans [45].

In this study, we measured Rad51 and Rad52 proteins in different tissues of mice after radiation. In the lungs, livers and kidneys of mice, the expression of Rad51 was highest in the mice that received the pCMV-HA-*pprI* plasmid. This suggests that PprI protein can promote the expression of Rad51 protein in mammals, especially on the 1st day after radiation, and that this increased expression of Rad51 protein occurred in different tissues. PprI protein may enhance the anti-radiation effect of mice by increasing the expression of Rad51 protein. We saw no obvious change of Rad52 protein expression in the lungs and livers of mice in all groups.

Here, we show that PprI protein can protect mice from radiation induced damage, and this protein holds great promise as a radioprotector. Further studies are warranted to determine the most effective application for PprI to be used in an effective strategy for the prevention and treatment of acute radiation injury.

## MATERIALS AND METHODS

### Animal group and radiation

Pure strain BALB/c mice (male, 6-8week of age, weighing between 16-20g) were obtained from the Experimental Animal Center of the Ministry of Medical of Suzhou University. All animal procedures were performed with approved protocols and in accordance with published recommendations for the proper use and care of laboratory animals. A total of 120 mice were randomly assigned to 5 groups, the resultant groups were the no irradiation control (*n* = 5), irradiation treatment with no plasmid (*n* = 25), irradiation treatment with the pCMV-HA empty vector (*n* = 25), irradiation treatment with the pCMV-HA-*pprI* gene (*n* = 25), and the mortality observed group (*n* = 40). The no irradiation control group received mock irradiation treatment. Mice were total body irradiated by ^60^Co γ ray at a dose rate was 2 Gy/min; absorbed dose was 6Gy. The mice were placed in aseptic rooms after radiation, and 5 of the mice in each group were sacrificed 1, 7, 14, 28, and 35 days post-irradiation. The mortality observed group contained 10 from each treatment group.

### Gene transfection

The plasmids were transfected by *in vivo* electroporation transfection into the mice 24 hours before irradiation. We injected the plasmid at a concentration of 60ug/60ul to the femoral muscle of mice. One minute later, we placed the electrode clamps of 384 type on both sides of the injection point and applied 8 pulses at 200V/cm strength, 20 msec duration, and 1HZ frequency using the ECM830 *in vivo* electroporation instrument (BTX co., American).

### Mortality rate of mice

We monitored the mice daily up to 30th days of radiation for signs of mortality and calculated the mortality rate (%).

### Counting and classification of peripheral blood cells

At 1, 7, 14, 28, and 35 days after radiation, five mice in each group were euthanized. Blood was sampled from the mice eyes and anti-coagulated by EDTA. Next, we determined the total number of white blood cells, red blood cells, platelets and the lymphocyte-to-white blood cell ratio using a blood analyzer (Shenzhen Mindray Bio-Medical Electronics Company, China).

### Apoptosis detection

Apoptotic cells were differentiated from viable or necrotic ones by combined application of Annexin V-FITC and propidium iodide (PI) (Invitrogen co., American). In brief, on the 1, 7, 14, 28, and 35 days after radiation, five mice in each group were euthanized. Spleen, thymuses and femurs were dissected from five mice in each group. We used the spleen and thymuses to prepare lymphocyte single cell suspensions with 2mL ice-cold phosphate buffered saline (PBS) using the comb scraping method. The marrow cavities of femurs were washed with 1mL PBS to prepare lymphocyte single cell suspensions. We centrifuged the single cell suspension, washed twice with PBS, discarded the supernatants, added 3mL Tris-NH4Cl(PH7.2), stirred, let stand 5min to lyse the red blood cells and added PBS to 10mL to stop the lysis. The samples were again centrifuged, supernatants were discarded, and cells were resuspended cells in AnnexinV-binding buffer at a concentration of 1 × 106 cells/mL. One hundred microliters of the solution was transferred to a 5-mL culture tube and then 5 μL of Annexin V and 1 μL of PI were added. The cells were gently vortexed and incubated for 15 min at room temperature (25 °C) in the dark. Four hundred microliters of 1×annexion-binding buffer was added to each tube and analyzed by flow cytometry (Beckman company, American) within 1 h. The results was expressed by apoptosis rates (%).

### Western blot analysis

Femoris muscle, lung, liver, and kidney tissue were dissected from five mice in each group at 1, 7, 14, 28, and 35 days after radiation. Tissues were cut into small pieces, and cell lysis solution with phenylmethylsulfonyl fluoride (PMSF) was added to 5 ml/g. Samples were incubated on ice in the glass homogenate for 30 minutes, then centrifuged at 10,000 rpm (centrifugal radius 10 cm) for 10 minutes at 4°C. The supernatant was transferred into a new tube, and stored at -80°C. We detected the protein concentration by the bicinchoninic acid (BCA) method and performed SDS-polyacrylamide gel electrophoresis (SDS-PAGE) and Western blot analyses. Samples (50ug, determined by BCA method) were separated on a 5% gel (containing 1.4 mL deionized water, 0.33 mL 30% acrylamide solution, 2.5 mL 1 mol Tris - HCl (pH 6.8), 0.02 mL 10% sodium dodecyl sulfate, 0.02 mL 10% ammonium sulfate, 0.002 mL tetramethyl ethylenediamine) with a 12% stacking gel (containing 3.3 mL deionized water, 4.0 mL 30% acrylamide solution, 2.5 mL 1.5 mol/L Tris - HCl (pH 8.8), 0.1 mL 10% sodium dodecyl sulfate, 0.1 mL 10% ammonium sulfate, and 0.004 mL tetramethyl ethylenediamine). The protein was separated at 80 V for 30 min, and at 100 V for 100 min. We transferred the proteins to the membranes for 2 hours. Membranes were incubated in TBS containing 5% skimmed milk for 1.5h. Blots were incubated overnight at 4°C in primary antibody solution (diluted 1:1,000 in TBS). Antibodies used were mouse polyclonal anti-HA, rabbit polyclonal anti-Rad51, rabbit polyclonal anti-Rad52, mouse monoclonal anti-GAPDH (Santa Cruz Biotechnology, Santa Cruz, CA), mouse monoclonal anti-beta-actin (Sigma Aldrich, St. Louis, MO). After washing three times for 10 min with TBS containing 0.05% Tween (TBST), blots were incubated in secondary antibody solution (diluted 1:5,000 in TBST) for 1 hour at 20°C. Antibodies used were goat anti-rabbit IgG (H+L) and goat anti-mouse IgG (H+L) (Santa Cruz Biotechnology, Santa Cruz, CA). After washing three times for 10 min, the signals of proteins were visualized using the electrochemiluminescence (ECL) kit (Pierce co., American). After exposure, development, and fixing, the results were scanned, and quantification was performed using Quantity One analysis software.

### Statistical analysis

Dates are shown as mean ± S.E.M. (standard error of the mean). Statistical analyses were performed in SAS 8.0 software. Analysis of Variance was used to analyze the experimental data, and one-way ANOVA was used to analyze each group of the experimental data. Differences were considered significant when *P* < 0.05.

## References

[1] Blasius M, Sommer S, Hubscher U (2008). Deinococcus radiodurans: what belongs to the survival kit?. Critical reviews in biochemistry and molecular biology.

[2] Cox MM, Battista JR (2005). Deinococcus radiodurans - the consummate survivor. Nature reviews Microbiology.

[3] Slade D, Radman M (2011). Oxidative stress resistance in Deinococcus radiodurans. Microbiology and molecular biology reviews.

[4] Levin-Zaidman S, Englander J, Shimoni E, Sharma AK, Minton KW, Minsky A (2003). Ringlike structure of the Deinococcus radiodurans genome: a key to radioresistance?. Science (New York, NY).

[5] Minsky A (2003). Structural aspects of DNA repair: the role of restricted diffusion. Molecular microbiology.

[6] Frenkiel-Krispin D, Minsky A (2006). Nucleoid organization and the maintenance of DNA integrity in E. coli, B. subtilis and D. radiodurans. Journal of structural biology.

[7] Zahradka K, Slade D, Bailone A, Sommer S, Averbeck D, Petranovic M, Lindner AB, Radman M (2006). Reassembly of shattered chromosomes in Deinococcus radiodurans. Nature.

[8] Daly MJ, Gaidamakova EK, Matrosova VY, Vasilenko A, Zhai M, Leapman RD, Lai B, Ravel B, Li SM, Kemner KM, Fredrickson JK (2007). Protein oxidation implicated as the primary determinant of bacterial radioresistance. PLoS biology.

[9] Fredrickson JK, Li SM, Gaidamakova EK, Matrosova VY, Zhai M, Sulloway HM, Scholten JC, Brown MG, Balkwill DL, Daly MJ (2008). Protein oxidation: key to bacterial desiccation resistance?. The ISME journal.

[10] Slade D, Lindner AB, Paul G, Radman M (2009). Recombination and replication in DNA repair of heavily irradiated Deinococcus radiodurans. Cell.

[11] White O, Eisen JA, Heidelberg JF, Hickey EK, Peterson JD, Dodson RJ, Haft DH, Gwinn ML, Nelson WC, Richardson DL, Moffat KS, Qin H, Jiang L (1999). Genome sequence of the radioresistant bacterium Deinococcus radiodurans R1. Science (New York, NY).

[12] Kobayashi Y, Watanabe H, Kikuchi M, Narumi I (2000). Effect of the space environment on the induction of DNA-repair related proteins and recovery from radiation damage. Advances in space research.

[13] Narumi I, Satoh K, Kikuchi M, Funayama T, Kitayama S, Yanagisawa T, Watanabe H, Yamamoto K (1999). Molecular analysis of the Deinococcus radiodurans recA locus and identification of a mutation site in a DNA repair-deficient mutant, rec30. Mutation research.

[14] Gutman PD, Fuchs P, Ouyang L, Minton KW (1993). Identification, sequencing, and targeted mutagenesis of a DNA polymerase gene required for the extreme radioresistance of Deinococcus radiodurans. Journal of bacteriology.

[15] Funayama T, Narumi I, Kikuchi M, Kitayama S, Watanabe H, Yamamoto K (1999). Identification and disruption analysis of the recN gene in the extremely radioresistant bacterium Deinococcus radiodurans. Mutation research.

[16] Earl AM, Mohundro MM, Mian IS, Battista JR (2002). The IrrE protein of Deinococcus radiodurans R1 is a novel regulator of recA expression. Journal of bacteriology.

[17] Gao G, Tian B, Liu L, Sheng D, Shen B, Hua Y (2003). Expression of Deinococcus radiodurans PprI enhances the radioresistance of Escherichia coli. DNA repair.

[18] Pan J, Wang J, Zhou Z, Yan Y, Zhang W, Lu W, Ping S, Dai Q, Yuan M, Feng B, Hou X, Zhang Y, Ma R (2009). IrrE, a global regulator of extreme radiation resistance in Deinococcus radiodurans, enhances salt tolerance in Escherichia coli and Brassica napus. PloS one.

[19] Zhou Z, Zhang W, Chen M, Pan J, Lu W, Ping S, Yan Y, Hou X, Yuan M, Zhan Y, Lin M (2011). Genome-wide transcriptome and proteome analysis of Escherichia coli expressing IrrE, a global regulator of Deinococcus radiodurans. Molecular bioSystems.

[20] Arpin D, Perol D, Blay JY, Falchero L, Claude L, Vuillermoz-Blas S, Martel-Lafay I, Ginestet C, Alberti L, Nosov D, Etienne-Mastroianni B, Cottin V, Perol M, Guerin JC, Cordier JF, Carrie C (2005). Early variations of circulating interleukin-6 and interleukin-10 levels during thoracic radiotherapy are predictive for radiation pneumonitis. Journal of clinical oncology.

[21] Hart JP, Broadwater G, Rabbani Z, Moeller BJ, Clough R, Huang D, Sempowski GA, Dewhirst M, Pizzo SV, Vujaskovic Z, Anscher MS (2005). Cytokine profiling for prediction of symptomatic radiation-induced lung injury. International journal of radiation oncology, biology, physics.

[22] Hua Y, Narumi I, Gao G, Tian B, Satoh K, Kitayama S, Shen B (2003). PprI: a general switch responsible for extreme radioresistance of Deinococcus radiodurans. Biochemical and biophysical research communications.

[23] Lu H, Chen H, Xu G, Shah AM, Hua Y (2012). DNA binding is essential for PprI function in response to radiation damage in Deinococcus radiodurans. DNA repair.

[24] Bettan M, Emmanuel F, Darteil R, Caillaud JM, Soubrier F, Delaere P, Branelec D, Mahfoudi A, Duverger N, Scherman D (2000). High-level protein secretion into blood circulation after electric pulse-mediated gene transfer into skeletal muscle. Molecular therapy.

[25] Blomberg P, Eskandarpour M, Xia S, Sylven C, Islam KB (2002). Electroporation in combination with a plasmid vector containing SV40 enhancer elements results in increased and persistent gene expression in mouse muscle. Biochemical and biophysical research communications.

[26] Dean DA (2013). Cell-specific targeting strategies for electroporation-mediated gene delivery in cells and animals. The Journal of membrane biology.

[27] Mir LM, Bureau MF, Gehl J, Rangara R, Rouy D, Caillaud JM, Delaere P, Branellec D, Schwartz B, Scherman D (1999). High-efficiency gene transfer into skeletal muscle mediated by electric pulses. Proceedings of the National Academy of Sciences of the United States of America.

[28] Bureau MF, Gehl J, Deleuze V, Mir LM, Scherman D (2000). Importance of association between permeabilization and electrophoretic forces for intramuscular DNA electrotransfer. Biochimica et biophysica acta.

[29] Vilquin JT, Kennel PF, Paturneau-Jouas M, Chapdelaine P, Boissel N, Delaere P, Tremblay JP, Scherman D, Fiszman MY, Schwartz K (2001). Electrotransfer of naked DNA in the skeletal muscles of animal models of muscular dystrophies. Gene therapy.

[30] Satkauskas S, Andre F, Bureau MF, Scherman D, Miklavcic D, Mir LM (2005). Electrophoretic component of electric pulses determines the efficacy of in vivo DNA electrotransfer. Human gene therapy.

[31] Markelc B, Tevz G, Cemazar M, Kranjc S, Lavrencak J, Zegura B, Teissie J, Sersa G (2012). Muscle gene electrotransfer is increased by the antioxidant tempol in mice. Gene therapy.

[32] Markelc B, Skvarca E, Dolinsek T, Kloboves VP, Coer A, Sersa G, Cemazar M (2015). Inhibitor of endocytosis impairs gene electrotransfer to mouse muscle in vivo. Bioelectrochemistry (Amsterdam, Netherlands).

[33] Peng B, Zhao Y, Lu H, Pang W, Xu Y (2005). In vivo plasmid DNA electroporation resulted in transfection of satellite cells and lasting transgene expression in regenerated muscle fibers. Biochemical and biophysical research communications.

[34] Dainiak N, Waselenko JK, Armitage JO, MacVittie TJ, Farese AM (2003). The hematologist and radiation casualties. Hematology American Society of Hematology Education Program.

[35] Sanzari JK, Wan XS, Krigsfeld GS, Wroe AJ, Gridley DS, Kennedy AR (2013). The Effects of Gamma and Proton Radiation Exposure on Hematopoietic Cell Counts in the Ferret Model. Gravitational and space research.

[36] Sanzari JK, Wan XS, Wroe AJ, Rightnar S, Cengel KA, Diffenderfer ES, Krigsfeld GS, Gridley DS, Kennedy AR (2013). Acute hematological effects of solar particle event proton radiation in the porcine model. Radiation research.

[37] Wilkins RC, Kutzner BC, Truong M, McLean JR (2002). The effect of the ratio of CD4+ to CD8+ T-cells on radiation-induced apoptosis in human lymphocyte subpopulations. International journal of radiation biology.

[38] Stahnke K, Mohr A, Liu J, Meyer LH, Karawajew L, Debatin KM (2004). Identification of deficient mitochondrial signaling in apoptosis resistant leukemia cells by flow cytometric analysis of intracellular cytochrome c, caspase-3 and apoptosis. Apoptosis.

[39] Krasin F, Hutchinson F (1977). Repair of DNA double-strand breaks in Escherichia coli, which requires recA function and the presence of a duplicate genome. Journal of molecular biology.

[40] Fujimori A, Tachiiri S, Sonoda E, Thompson LH, Dhar PK, Hiraoka M, Takeda S, Zhang Y, Reth M, Takata M (2001). Rad52 partially substitutes for the Rad51 paralog XRCC3 in maintaining chromosomal integrity in vertebrate cells. The EMBO journal.

[41] Valerie K, Povirk LF (2003). Regulation and mechanisms of mammalian double-strand break repair. Oncogene.

[42] de la Tour CB, Passot FM, Toueille M, Mirabella B, Guerin P, Blanchard L, Servant P, de Groot A, Sommer S, Armengaud J (2013). Comparative proteomics reveals key proteins recruited at the nucleoid of Deinococcus after irradiation-induced DNA damage. Proteomics.

[43] Ngo KV, Molzberger ET, Chitteni-Pattu S, Cox MM (2013). Regulation of Deinococcus radiodurans RecA protein function via modulation of active and inactive nucleoprotein filament states. The Journal of biological chemistry.

[44] Sung P, Krejci L, Van Komen S, Sehorn MG (2003). Rad51 recombinase and recombination mediators. The Journal of biological chemistry.

[45] New JH, Sugiyama T, Zaitseva E, Kowalczykowski SC (1998). Rad52 protein stimulates DNA strand exchange by Rad51 and replication protein A. Nature.

[46] Symington LS (2002). Role of RAD52 epistasis group genes in homologous recombination and double-strand break repair. Microbiology and molecular biology reviews.

